# Baseline Characteristics of a Cluster Randomized Controlled Trial Targeting Hand Hygiene in Primary Healthcare in Burkina Faso and Mali

**DOI:** 10.3389/ijph.2025.1608406

**Published:** 2025-05-21

**Authors:** Anaïs Galli, Mirko S. Winkler, Jan Hattendorf, Max N. D. Friedrich, Issa Bagayogo, Aboubacar Ballo, Carola Bänziger, Hassane Dembélé, Mamadou Sory Keita, Maryna Peter, Alimata Ousséni Tall, Jürg Utzinger, Branwen Nia Owen

**Affiliations:** ^1^ University of Basel, Basel, Switzerland; ^2^ Swiss Tropical and Public Health Institute (Swiss TPH), Allschwil, Switzerland; ^3^ Ranas, Zurich, Switzerland; ^4^ Terre des hommes, Bamako, Mali; ^5^ University of Applied Sciences and Arts Northwestern Switzerland, Muttenz, Switzerland; ^6^ Division Hygiéne Publique, Direction Régionale de la Santé, Ségou, Mali; ^7^ Terre des hommes, Ouagadougou, Burkina Faso

**Keywords:** behavior change, infection prevention and control, handwashing, protracted conflict, water, sanitation and hygiene (WASH)

## Abstract

**Objectives:**

This study presents baseline characteristics of a cluster randomized controlled trial (cRCT) on hand hygiene in primary healthcare in Burkina Faso and Mali, addressing data gaps on hand hygiene practices in these settings.

**Methods:**

We implemented a two-arm cRCT in 48 primary healthcare facilities. Baseline data were collected (January–June 2023), followed by covariate-constrained randomization. We conducted covert hand hygiene observations, hand-rinse sampling for *Escherichia coli* detection, and a survey on behavioral factors among healthcare workers. The primary outcome is observed handwashing rate.

**Results:**

Baseline data included 309 healthcare workers. Trial arms were balanced in hand hygiene adherence, behavioral factors, and *E. coli* contamination. Hand hygiene adherence was low (12%). *E. coli* contamination was very high in Burkina Faso (76%) and considerable in Mali (23%). Participants had a high intention to wash their hands (93%) but only a quarter could name all moments for hand hygiene.

**Conclusion:**

Poor hand hygiene and *E. coli* contamination in our setting may heighten nosocomial infection risks. Interventions should address knowledge and build on high intentions to perform hand hygiene.

## Introduction

Hand hygiene is the most important measure for preventing the transmission of harmful microorganisms in healthcare [1]. Particularly in conflict settings, where the population is vulnerable and healthcare systems are weak or overburdened, these measures are critical to protect against preventable infections, such as diarrheal diseases, respiratory tract infections, and healthcare-associated infections [2]. Despite the importance of hand hygiene in crisis-affected populations, infrastructure and consumables are often lacking, and the changing environment and mental health burden can influence hand hygiene practices [3, 4]. Protracted conflicts have become more frequent in recent years, especially in sub-Saharan Africa [5]. Research investigating hand hygiene practices in healthcare settings affected by protracted conflict is scarce and needs to be strengthened to better understand the needs of healthcare workers and patients [6–8].

Primary healthcare is an integral part of health systems to achieve universal health coverage, while being accessible to all in an equitable manner [9]. Yet, studies on hand hygiene in healthcare mainly focus on hospital settings, predominantly in high-income countries [10, 11]. In West African countries affected by conflict, data are scarce and only consider hospital settings, in which hand hygiene adherence is very low with about 8%–20% [8, 12]. In these contexts, knowledge about hand hygiene practices in primary healthcare is crucial for local decision makers to coordinate efforts for infection prevention and control in areas where resources are limited and the population is extremely vulnerable.

Hands4health is a research project with the aim of improving water, sanitation and hygiene (WASH) infrastructure and practices in primary healthcare facilities without direct indoor water supply access in Burkina Faso and Mali and in schools in Nigeria and Palestine [13]. In this project, we are implementing a cluster randomized controlled trial (cRCT), which seeks to evaluate the effectiveness of a multi-component hand hygiene intervention. With the aim of describing hand hygiene practices in an under-researched setting, we present baseline characteristics of primary healthcare facilities, by study arm and country, of the on-going cRCT in the hands4health project.

## Methods

### Study Setting and Population

**FIGURE 1:**
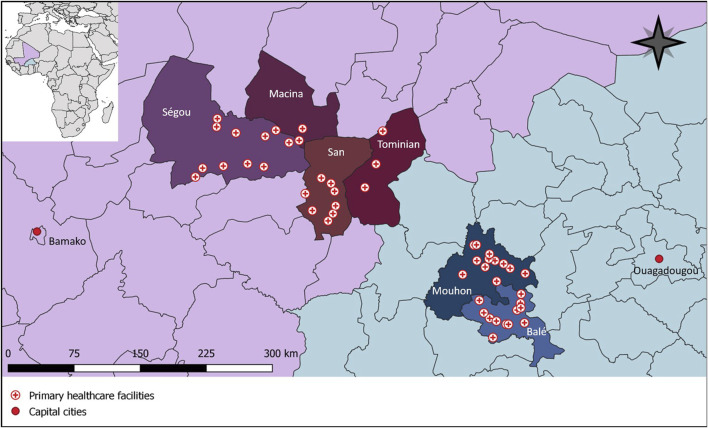
Primary healthcare facilities participating in a cluster randomized controlled trial in Burkina Faso and Mali with baseline data collected from January to June 2023 (hands4health, Burkina Faso and Mali, 2023).

In Burkina Faso and Mali, collaborating non-governmental organizations act with local health authorities as implementation partners with the overarching aim of strenghthening the health system. The cRCT was conducted in the provinces of Balé and Mouhoun in Burkina Faso and in the region of Ségou in the circles of Macina, San, Ségou, and Tominian in Mali (Figure 1) [13]. Both countries are politically instable and suffer from an insurgence of armed extremist groups [14, 15]. We selected these regions with our local implementation partners because (i) they previously completed successful collaborations with the regional health authorities, (ii) humanitarian access was still possible, and (iii) healthcare facilities were in need of water infrastructure and were prone to be overburdened due to a high number of internally displaced persons [13]. In both countries, we focused on rural primary healthcare facilities. These facilities mainly serve the community by providing maternal and neonatal care, vaccination programs, treatment of common diseases such as malaria and diarrhea, and implementing child nutrition programs. All of the facilities targeted in this cRCT were previously assessed with a Facility and Evaluation Tool for WASH in Institutions (FACET) and lacked direct indoor water supply access [16]. Most of these facilities had a water source on their premises and people had to fill buckets with taps which were then used within the building as handwashing stations. These buckets had several drawbacks: (i) to close the tap, people had to touch them again with their clean hands; (ii) many of the buckets were broken because the plastic did not withstand the heat and sun exposure; (iii) the bowl that collected gray water often overflowed and made the surroundings unhygienic; and (iv) buckets were often empty because there was not enough time or capacity to refill them. Surface technicians are responsible for refilling the buckets. They are volunteers with no job description or adequate salary. Consequently, surface technicians often lack qualifications, motivation, and willingness to stay at a healthcare facility for a long time [17]. Difficulties in accessing WASH services additionally contribute to the turnover of technical healthcare staff from rural to peri-urban and urban areas [18]. According to an infrastructure assessment conducted by project partners, at baseline, the median number of handwashing stations per facility was 2.5 in Burkina Faso and 4 in Mali. In both countries, all assessed facilities stocked alcohol-based handrub. However, in Burkina Faso 20% of consultation rooms and 35% of maternity wards lacked a handwashing station and alcohol-based handrub was missing in 95% of consultation rooms and absent in all maternity wards. In Mali, 25% of consultation rooms and 8% of maternity wards did not have any handwashing station, while alcohol-based handrub was absent in 46% of consultation rooms and 37% of maternity wards.

### Study Design

**FIGURE 2:**
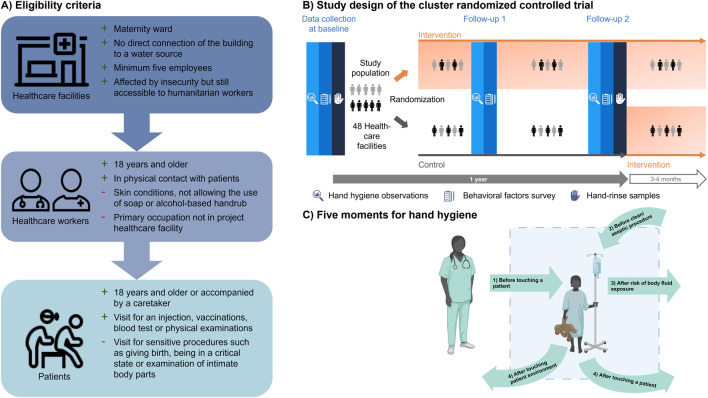
**(A)** Eligibility criteria with reasons for inclusion (+) and exclusion (−) for primary healthcare facilities, healthcare workers and patients who were present during the covert observations. Icons are from healthicons.org; **(B)** Study population of a cluster randomized controlled trial that includes handwashing observations, self-reported survey on behavioral factors and hand-rinse samples in Burkina Faso and Mali; **(C)** The WHO five moments for hand hygiene used to define handwashing opportunities during the covert hand hygiene observations in Burkina Faso and Mali in 2023 [11]. Created with BioRender.com (hands4health, Burkina Faso and Mali, 2023).

The selection process of healthcare facilities included three steps. First, the implementation partners characterized healthcare facilities using FACET [16]. Second, eligibility of the healthcare facilities was assessed with previously established eligibility criteria for healthcare facilities (Figure 2A). Finally, the implementation partners selected 24 health facilities in each country that were most likely to remain accessible within 12 months. In the selected facilities, healthcare workers were chosen according to the eligibility criteria shown in Figure 2A. In addition, patient encounters during covert handwashing observations of healthcare workers had to fulfill a set of previously defined eligibility criteria to ensure patient privacy (Figure 2A) [13].

Our cRCT comprised two study arms (Figure 2B) to assess the effectiveness of a multi-component hand hygiene intervention on improving hygiene-related behaviors and underlying behavioral factors of healthcare workers and patients over a 12-month period. In both countries, the same study design was employed. The intervention was implemented after the baseline data collection and consisted of hardware, behavior change, and management and monitoring [19] (for more information see Supplementary Material). Due to the nature of the intervention, blinding of participants and observers was not feasible. However, laboratory personnel working on microbial hand-rinse samples were blinded. Results of this cRCT are reported according to the Consolidated Standards of Reporting Trials (CONSORT) extension for cRCTs [20].

### Data Collection of Baseline Characteristics

We used three different tools to collect baseline data from January to June 2023 (Figure 2B). Details of the study methodology have been described elsewhere [13]. In brief, the following tools were employed:

1) face-to-face survey on behavioral factors;
2) covert structured hand hygiene observations; and
3) hand-rinse samples.

Survey data and hand-rinse samples were collected by well-trained implementation partners. For the observations, we trained district health authorities in Burkina Faso and personnel from a different department of the implementation partners in Mali. Observers were known to the study participants in a different function that was not related to any of the project activities. Observers in both countries visited healthcare facilities regularly for so-called supervisions. During those routine supervisions, they observed regular care practices, without any specific focus on hand hygiene, in order to assure quality of care in the healthcare facilities and therefore had free access to all patient areas. By training observers who were not involved in our project, we aimed to link their presence in the facilities to their usual supervision duties, and hence, minimize the Hawthorne effect on the hand hygiene practices [21]. Instead of their usual supervisions, the observers only observed in an Open Data Kit (ODK) form that was installed on their smartphone and adapted from the WHO observation form [22]. Additionally, observations always preceded the other data collection methods to avoid that study participants consciously link them to the survey and hand-rinse samples.

### Primary and Secondary Outcomes

The primary outcome of this cRCT is the observed handwashing rate of healthcare workers. The rate of handwashing is defined as the number of correct hand hygiene actions, namely handwashing with water and soap, the use of alcohol-based handrub or proper use of gloves, divided by the number of handwashing opportunities that occurred during an observation period of 1 hour. Handwashing opportunities were defined according to the WHO five moments for hand hygiene (Figure 2C) [11].

Secondary outcomes of this study are (i) self-reported hand hygiene practices, (ii) self-reported behavioral factors, and (iii) screening of healthcare workers hands for *Escherichia coli* during spot-checks. The self-reported variables were measured on a five-point Likert scale and then transformed to a score from zero to one [13]. *E. coli* were counted as colony-forming units (CFUs) with a lower detection limit of 3.5 CFUs and an upper detection limit of 1,050 CFUs per sample collected from both hands of the participants [23].

### Sample Size

To assure a statistical power of 81% at a two-sided significance level of 5% for the primary outcome of this cRCT, we ran a series of simulations assuming (i) an intra-cluster correlation coefficient of 0.15, (ii) six participants per healthcare facility, (iii) that participants should wash their hands correctly a total number of five times (Poisson distributed, mean = 5 and standard deviation = 2), considering the WHO moments for hand hygiene (Figure 2C) while they are being observed; and (iv) a difference of 15 percentage points in the handwashing rate between the two arms of the trial [24]. The simulations revealed that 10 facilities per arm were sufficient. Taking into account potential loss to follow-up, we enrolled 12 facilities per arm and country; hence, 48 facilities in total.

### Randomization

To minimize the risk of baseline imbalances, we applied restricted randomization for the allocation of facilities into the control and intervention arms after baseline data collection [25]. We used four constraints for finding potential allocation sequences: (i) the proportion of facilities with different water sources (source type and water shortages) had to be balanced; (ii) the difference in the mean proportion of people living within 5 km of the facilities’ catchment area had to be <10%; (iii) the difference in the proportion of safe facilities had to be <10%; and (iv) the difference in the mean proportion of correct handwashing during the WHO five moments for hand hygiene had to be <3%. We randomly chose one of 767,792 sequences satisfying all criteria in Burkina Faso and one of 248,574 sequences in Mali [13].

### Data Management and Analysis

Observational data, data on participants’ characteristics, and hand-rinse outcomes were collected using ODK Central (version 2022.3.1) on smartphones and tablets. Data analysis was conducted in R (version 4.1.3; R Foundation for Statistical Computing, Vienna, Austria) using the tidyverse package (version 2.0.0) (Wickham et al., 2019). We stratified our results by country and intervention-control arm.

## Results

### Enrolment and Baseline Characteristics

**FIGURE 3:**
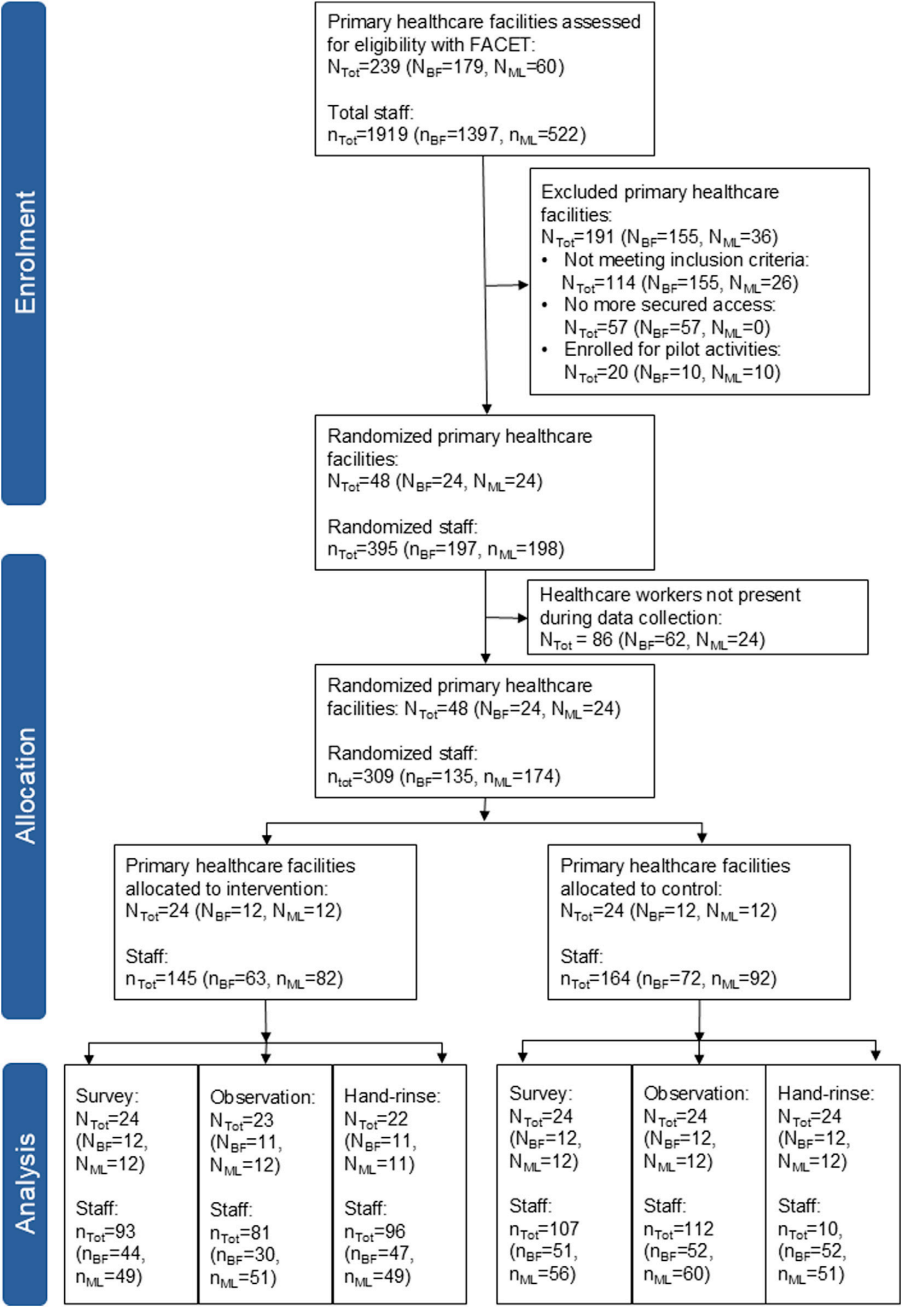
Consolidated Standards of Reporting Trials (CONSORT) flow diagram for the allocation of primary healthcare facilities in Burkina Faso (BF), Mali (ML) and both countries together (Tot) and the data collection (hands4health, Burkina Faso and Mali, 2023).

We screened 179 primary healthcare facilities in Burkina Faso and 60 in Mali with the FACET from September 2021 to December 2022 (Figure 3). We excluded 80% of the facilities as they did not meet the inclusion criteria, due to insecurity and because they were pilot facilities for our project. Finally, we enrolled 48 primary healthcare facilities for the baseline data collection with a total of 309 healthcare workers participating in at least one aspect of baseline data collection (Figure 3). Overall 90 healthcare workers participated in all three data collection tools (Burkina Faso, n = 51; Mali, n = 39).

After baseline data collection, the primary healthcare facilities were randomized into two study arms per country with 12 facilities per arm and country with a total of 145 participants in the intervention arm and 164 in the control arm (Figure 3). The facilities had an intra-cluster correlation coefficient of 0.04 in Burkina Faso and 0.11 in Mali for the primary outcome. Observation data of one healthcare facility in the intervention arm of Burkina Faso were lost due to transmission issues and hand-rinse samples in one intervention facility in each country could not be collected due to a lack of laboratory material.

### Demographic Characteristics of Healthcare Workers

Demographic characteristics collected in the self-reported survey were largely balanced across study arms. Across countries there were some notable differences (Table 1). The most common professional category in Burkina Faso was “nurses and midwives” (64%) and in Mali “medical support personnel” (54%) (Table 1). The educational level was higher in Burkina Faso, and fewer participants were married or cohabitating.

**TABLE 1:** Demographic characteristics of healthcare workers, stratified by trial arm and country (hands4health, Burkina Faso and Mali, 2023).

	Characteristic	Burkina Faso			Mali		
		Overal^a^ (n = 95)	Control^a^ (n = 51)	Intervention^a^ (n = 44)	Overall^a^ (n = 105)	Control^a^ (n = 56)	Intervention^a^ (n = 49)
Sex	Female	65 (68)	38 (75)	27 (61)	68 (65)	37 (66)	31 (63)
	Male	30 (32)	13 (25)	17 (39)	37 (35)	19 (34)	18 (37)
Age (years)	18–24	1 (1)	1 (2)	0 (0)	15 (14)	8 (14)	7 (14)
	25–49	92 (97)	49 (96)	43 (98)	82 (78)	46 (82)	36 (73)
	50+	2 (2)	1 (2)	1 (2)	8 (8)	2 (4)	6 (12)
Educational attainment	Completed primary school	26 (27)	15 (29)	11 (25)	62 (59)	30 (54)	32 (65)
	Completed secondary school	55 (58)	28 (55)	27 (61)	35 (33)	22 (39)	13 (27)
	Completed university or equivalent	14 (15)	8 (16)	6 (14)	8 (8)	4 (7)	4 (8)
Marital status	Married or cohabiting	61 (64)	36 (71)	25 (57)	91 (87)	47 (84)	44 (90)
	Single, divorced, separated or widowed	33 (35)	14 (27)	19 (43)	14 (13)	9 (16)	5 (10)
	Not declared	1 (1)	1 (2)	0 (0)	0 (0)	0 (0)	0 (0)
Profession	Medical doctor	1 (1)	0 (0)	1 (2)	3 (3)	1 (2)	2 (4)
	Nurses and midwifes	61 (64)	35 (69)	26 (59)	39 (37)	24 (43)	15 (31)
	Medical support personnel	33 (35)	16 (31)	17 (39)	57 (54)	29 (52)	28 (57)
	Other	0 (0)	0 (0)	0 (0)	6 (6)	2 (4)	4 (8)
Years of experience		7 (5, 10)	6 (4, 10)	8 (5, 11)	5 (2, 12)	4 (2, 9)	7 (2, 13)

^a^Numbers are N (%) or median (Q1, Q3).

### Hand Hygiene Practices of Healthcare Workers

#### Observation

**FIGURE 4:**
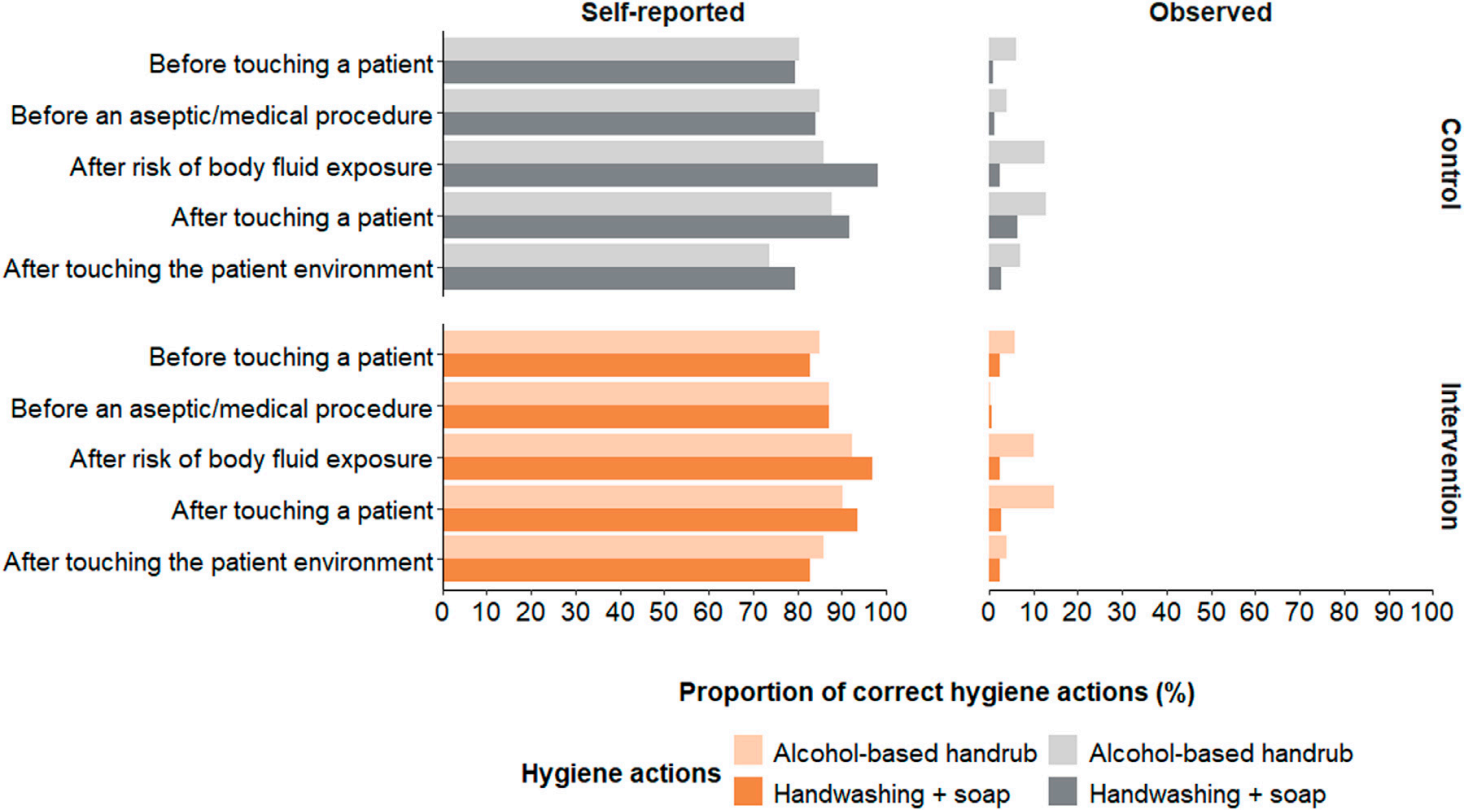
Comparison of self-reported and observed hand hygiene actions taken during the World Health Organization (WHO)’s five moments for hand hygiene. Displayed self-reported actions were reported as often or always performing the action (hands4health, Burkina Faso and Mali, 2023).

The proportion of observed correct hygiene actions (i.e., handwashing with soap, using alcohol-based handrub or proper glove use) was 12% overall (16% in Burkina Faso and 7% in Mali) with minor differences between intervention arms (Figure 4; Supplementary Table S1). Overall, healthcare workers in Burkina Faso performed most of their hand hygiene actions after body fluid exposure, with 26% of healthcare workers executing a hygiene action during this opportunity (Supplementary Table S1). In Mali, most actions took place after touching a patient, with 18% of healthcare workers completing a hygiene action after touching a patient. Alcohol-based handrub was used 3.3 times more often in Burkina Faso and 2.5 times more often in Mali than washing hands with water and soap.

#### Self-Reported

Self-reported hand hygiene was much higher than observed hand hygiene with more than 80% of the healthcare workers reporting handwashing with soap or use of alcohol-based handrub “often” or “almost always” during the WHO five moments for hand hygiene (Figure 4). In both countries, most reported to perform hand hygiene “often” or “almost always” after being exposed to body fluids (Supplementary Table S1). The preferred self-reported hand hygiene action was handwashing with soap for 65% of the healthcare workers in Burkina Faso and 58% in Mali.

### *E. coli* Contamination on Healthcare Workers’ Hands

**FIGURE 5:**
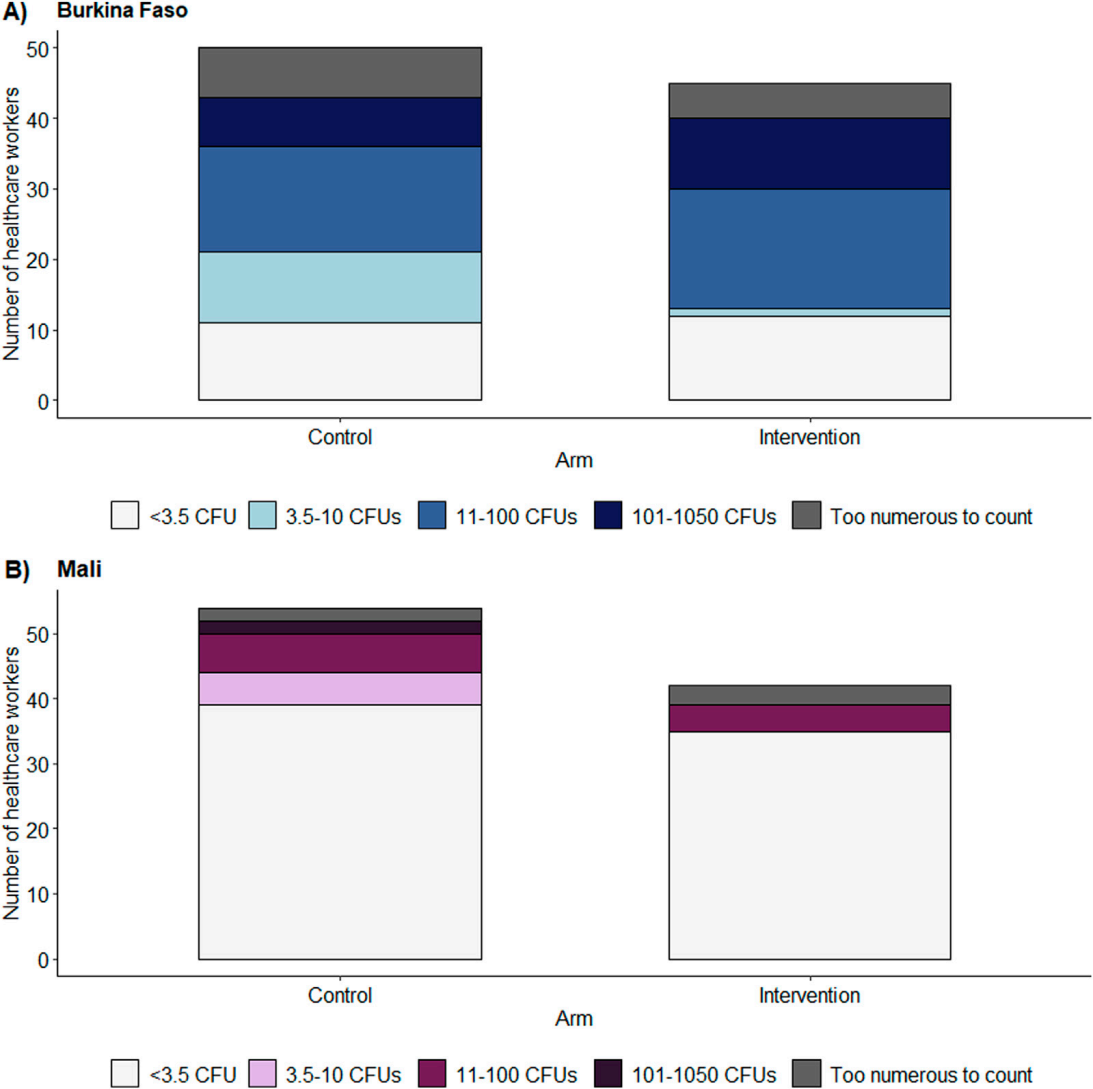
Bar plots of the number of colony-forming units of *Escherichia coli* on the hands of healthcare workers stratified according to intervention arms in **(A)** Burkina Faso and **(B)** Mali. As there are no available categories for *E. coli* contamination on healthcare worker’s hands, the World Health Organization (WHO)’s categories for drinking water risk assessment were used taking into account our detection limits (<3.5 colony-forming unit = low risk, 3.5-10 colony-forming units = intermediate risk, 11-100 colony-forming units = high risk, 101-1050 colony-forming units = very high risk) (WHO, 2022a) with a lower detection limit of 3.5 colony-forming units and an upper detection limit of 1,050 colony-forming units (hands4health, Burkina Faso and Mali, 2023).

*E. coli* was detected on 76% of tested participants’ hands in Burkina Faso and 23% in Mali (Supplementary Table S3; Figure 5). The majority of participants in Burkia Faso had a high risk contamination with 11-100 CFUs (34%). *E. coli* presence on healthcare workers’ hands was balanced between the trial arms and did not correlate with the mean proportion of correct hand hygiene actions taken during the observations (Figure 5). Daily tests for *E. coli* contamination of the water used for the hand rinse sampling remained all negative (<3.5 CFUs).

### Hygiene-Related Knowledge and Behavioral Factors

A quarter (24%) of participants could name all five WHO moments for hand hygiene. This proportion was higher in Mali (30%) than in Burkina Faso (16%) (Supplementary Tables S3, S4). The WHO handwashing steps were well demonstrated by most participants except for “palm to palm with fingers interlaced,” which was only shown by 26% of participants in Burkina Faso (Supplementary Table S5).

When asked about the five WHO moments for hand hygiene, most participants (93%) reported having an intention to wash their hands “often or always” in these moments. Similarly, most participants indicated that hand hygiene norms were high and that there were not many barriers to hand hygiene (Supplementary Table S6). More participants in Burkina Faso (35%) than in Mali (13%) reported that collecting water and constantly washing their hands during the five moments took a “big or extreme” effort (Supplementary Table S6).

## Discussion

We identified a severe lack of hand hygiene adherence during the five WHO moments for hand hygiene in primary healthcare facilities of Burkina Faso and Mali. In contrast, self-reported hand hygiene practices in both countries were 20-fold that of observed practices. *E. coli* contamination was common, being detected on three-quarters and a quarter of healthcare workers’ hands in Burkina Faso and Mali, respectively. This paper describes baseline results of an on-going cRCT which aims to evaluate the effectiveness of a multi-component hand hygiene intervention in primary healthcare facilities without direct water supply access. Our results here demonstrate that the trial arms were balanced with regard to baseline characteristics, hand hygiene adherence, and *E. coli* contamination. Consequently, our results indicate that the randomization process was successful and follow-up results will be comparable between trial arms.

To our knowledge, this paper presents the first published estimate of observed hand hygiene during the five moments for hand hygiene in primary care in Burkina Faso and Mali. Despite participants’ low perception of barriers for hand hygiene, the low level of adherence across the five moments for hand hygiene can partly be explained by a lack of infrastructure. For example, no maternity ward in Burkina Faso was equipped with alcohol-based handrub. However, considering that in Mali infrastructure availability was more prevalent, but hygiene practices were less frequent than in Burkina Faso, infrastructure availability cannot be the sole reason for low adherence. Our observed hand hygiene rates are similar to estimates gathered during observation studies in tertiary care. A recent review of global hand hygiene practices in healthcare estimated that low- and middle-income countries have an average hand hygiene adherence of less than 20% [26, 27]. Similarly, a study in Burkina Faso, investigating maternal and newborn care, reports handwashing before a patient examination for 21% of healthcare workers in primary care and 19% in referral hospitals [12]. In Mali, a study reported a hand hygiene compliance of 8% in a hospital setting, corroborating with our findings of a compliance of 7% [8]. Concerning the specific moments for hand hygiene, the moments “after touching a patient” and “after exposure to body fluids” were best adhered to in our study. Similar observations were made in a systematic review investigating hand hygiene practices in intensive care units internationally [26]. Both these most adhered moments for hand hygiene might break the transmission route of microorganisms from the patient to the healthcare worker. This may suggest that healthcare workers prioritize protecting themselves over protecting patients. Taken together, our findings on hand hygiene adherence in primary healthcare in Burkina Faso and Mali are in line with the limited data published thus far.

A substantial proportion of healthcare workers’ hands were contaminated with *E. coli*. This finding is worrisome because the presence of *E. coli* serves as an indicator of fecal contamination [28]. There is a paucity of literature reporting *E. coli* screening on healthcare workers’ hands, especially in primary healthcare. Screening seems to take place mostly after hospital outbreaks of gram-negative bacteria to detect the source of the outbreak [29]. We identified one study, conducted in a tertiary care context in the United States that detected *E. coli* on 6.2% of healthcare workers’ hands [30]. These findings indicate a much lower proportion of *E. coli* contamination compared to our results. Additionally, studies in households in Bangladesh, Tanzania, and Zimbabwe found a high level of *E. coli* contamination in soil and on surfaces [31–33]. Tanzanian mothers’ hand contamination was related to normal household activities such as cooking and not exclusively to defecating or cleaning a child’s feces [34]. Hence, other routes of potential *E. coli* transmission should be considered, such as soil in healthcare facilities. To conclude, there is not enough publicly accessible data about the proportion of healthcare workers’ hands contaminated with *E. coli* and contamination might have multiple origins. Hence, we recommend further research investigating the suitability of *E. coli* as an indicator organism for hand hygiene in healthcare and transparent reporting practices.

### Strengths and Limitations

The baseline results at the outset of a cRCT demonstrate some noteworthy strengths. First, by combining multiple measurement methods, we aimed to increase our understanding of hand hygiene practices and their determinants, which are usually very difficult to assess [35]. Second, by randomizing the clusters after the baseline data collection we ensured that the most important baseline characteristics of clusters are balanced. Third, actively involving local authorities and implementation partners early on in this trial allowed us to design the study and tailor intervention to the communities’ needs. Finally, our use of covert observations with observers that usually frequent the investigated facilities minimized the Hawthorne effect. To address ethical concerns of covert observations [11, 36], we had in-depth discussions with our local partners, authorities, and local healthcare workers, identifying the best way to not violate any trust. However, future studies measuring the Hawthorne effect in primary care with overt and covert observations would be highly valuable. The magnitude of the effect has been found to vary across hospital wards, but data of the effect at primary healthcare level remain scarce [36].

This study is not without limitations. First, staff presence at the healthcare facilities was highly volatile, with staff often away for trainings, not working regularly, or moving because of deteriorating security situation. Consequently, the number of participants involved in all three data collection tools remained low. Second, due to a data transmission issue, we lost hand hygiene observation data from one healthcare facility in the intervention group and due to a lack of laboratory material we could not collect hand-rinse samples in two facilities of the intervention group. Third, we used a proxy for infection rate by measuring hand hygiene as our primary outcome. We are aware that directly measuring infection rates would have produced results that are easier to understand and use for advocacy. However, considering that most hygiene-related infections not only originate in healthcare settings but also in the community, solely collecting data in healthcare facilities would not have been sufficient and would have required a considerably larger sample size. Fourth, the five moments for hand hygiene have been previously critiqued to not fully capture the realities of settings with limited resources and overcrowding [37, 38]. Despite vigorous training, observers in this study sometimes had to report non-observable actions because patient zones were not clearly visible, multiple people were working on a patient or visibility was generally bad. Despite the limitations of the five moments for hand hygiene, we decided to use this method as it remains the recommended method by the WHO and allows comparability with other studies and across contexts [11]. Finally, we are well aware of the desirability bias present in the self-reported survey. This bias has previously been reported in hand hygiene assessments during COVID-19 by a systematic literature review [39]. Due to the survey-data driven behavior change campaign that the intervention group received and to better understand perceptions of participants, we decided to still report the results [13, 40].

### Conclusions

The inadequate hand hygiene practices observed in the two study countries are consistent with prior studies in hospitals in both countries. We are not aware of published estimates in primary healthcare settings. The high proportion of *E. coli* contamination on healthcare workers’ hands indicates a high risk of bacterial transmission, but needs to be regarded with precaution as the use of *E. coli* as a hand hygiene indicator remains to be further established. We highly recommend interventions targeting hand hygiene practices and WASH infrastructure in primary healthcare facilities to effectively prevent infection.

## Abbreviations

CFU, Colony-forming unit; CONSORT, Consolidated Standards of Reporting Trials; cRCT, Cluster randomized controlled trial; FACET, Facility Evaluation Tool for WASH in Institutions; ODK, Open Data Kit; RANAS, Risks, Attitudes, Norms, Abilities, and Self-regulation; WASH, Water, sanitation and hygiene; WASH FIT, Water and Sanitation for Health Facility Improvement Tool; WHO, World Health Organization.
