# Supplementary material for: Baseline Characteristics of a Cluster Randomized Controlled Trial Targeting Hand Hygiene in Primary Healthcare in Burkina Faso and Mali
Source: Int J Public Health. 2025 May 21;70:1608406. doi: 10.3389/ijph.2025.1608406 (PMC12133522; doi:10.3389/ijph.2025.1608406)
Supplement: Supplementary file 1 [file DataSheet1.docx]

# Supplementary material

Multi-component hand hygiene intervention

Based on the different local needs in the study countries, our project partners designed a multi-component hand hygiene intervention. The intervention consisted of (i) the installation of novel handwashing station(s) that recycle water by gravity (Gravit’eau), (ii) a behavior change campaign tailored to health care workers, (iii) implementation of the Water and Sanitation for Health Facility Improvement Tool (WASH FIT), (iv) local capacity building for drinking water quality monitoring and chlorination and (v) support for preventive maintenance and management of WASH infrastructure in health care facilities. Facilities of the control group that were still accessible and in need of hygiene infrastructure received a handwashing station, the behavior change campaign and a WASH FIT implementation starting in April 2024.

# Supplementary tables

**Supplementary Table 1:** *Observed and self-reported hand hygiene actions in Burkina Faso and Mali categorized according to WHO moment for hand hygiene*.

| **WHO moment** | **Action^1^** | **Burkina Faso observed** | | **Mali observed** | | **Burkina Faso self-reported** | | | **Mali self-reported** | | |
| --- | --- | --- | --- | --- | --- | --- | --- | --- | --- | --- | --- |
|  |  | *Contr.^2^* | *Int.^2^* | *Contr.^2^* | *Int.^2^* | *Contr.^2^*  (N=51) | *Int.^2^*  (N=44) | *Contr.^2^*  (N=56) | | *Int.^2^*  (N=49) |  |
| Opportunities^3^ |  | 263 | 194 | 72 | 72 | NA | NA | NA | | NA |  |
| **Before touching a patient** | HR | 34 (13) | 20 (10) | 0 (0) | 2 (3) | 41 (80) | 37 (84) | 45 (80) | | 42 (86) |  |
|  | HWS | 5 (2) | 7 (4) | 0 (0) | 1 (1) | 39 (76) | 37 (84) | 46 (82) | | 40 (82) |  |
|  | G | 5 (2) | 8 (4) | 0 (0) | 0 (0) | NA | NA | NA | | NA |  |
| Opportunities^3^ |  | 147 | 110 | 103 | 92 | NA | NA | NA | | NA |  |
| **Before an aseptic procedure** | HR | 7 (5) | 0 (0) | 2 (2) | 1 (1) | 43 (84) | 38 (86) | 48 (86) | | 43 (88) |  |
|  | HWS | 2 (1) | 1 (1) | 0 (0) | 0 (0) | 44 (86) | 38 (86) | 46 (82) | | 43 (88) |  |
|  | G | 1 (1) | 2 (2) | 1 (1) | 0 (0) | NA | NA | NA | | NA |  |
| Opportunities^3^ |  | 125 | 119 | 101 | 94 | NA | NA | NA | | NA |  |
| **After body fluid exposure** | HR | 22 (18) | 24 (20) | 2 (4.17) | 1 (1) | 42 (82) | 40 (91) | 50 (89) | | 46 (94) |  |
|  | HWS | 2 (2) | 8 (7) | 0 (0) | 0 (0) | 50 (98) | 43 (98) | 55 (98) | | 47 (96) |  |
|  | G | 0 (0) | 0 (0) | 1 (2.78) | 1 (1) | NA | NA | NA | | NA |  |
| Opportunities^3^ |  | 221 | 173 | 77 | 69 | NA | NA | NA | | NA |  |
| **After touching a patient** | HR | 42 (19) | 25 (14) | 6 (8) | 11 (16) | 45 (88) | 40 (91) | 49 (88) | | 44 (90) |  |
|  | HWS | 7 (3) | 6 (3) | 6 (8) | 0 (0) | 46 (90) | 41 (93) | 52 (93) | | 46 (94) |  |
|  | G | 0 (0) | 0 (0) | 0 (0) | 1 (1) | NA | NA | NA | | NA |  |
| Opportunities^3^ |  | 158 | 107 | 32 | 19 | NA | NA | NA | | NA |  |
| **After touching the patient environment** | HR | 12 (8) | 4 (4) | 0 (0) | 1 (5) | 40 (78) | 35 (80) | 39 (70) | | 45 (92) |  |
|  | HWS | 2 (1) | 4 (4) | 2 (6) | 0 (0) | 41 (80) | 34 (77) | 44 (79) | | 43 (88) |  |
|  | G | 0 (0) | 0 (0) | 0 (0) | 0 (0) | NA | NA | NA | | NA |  |

^1^ HR = alcohol-based handrub, HWS = handwashing with soap, G = proper glove use

^2^ Numbers are N (%)

^3^ Number of total observed hand hygiene opportunities for the corresponding WHO moment for hand hygiene.

#### **Supplementary Table 2:** Overview of number of health care workers with E. coli present on their hands and the quantity of Colony Forming Units (Colony Forming Units)

|  | **Burkina Faso (N=95)** | | | **Mali (N=96)** | | | |  |
| --- | --- | --- | --- | --- | --- | --- | --- | --- |
|  | *Overall^1^* | *Contr. ^1^* | *Int. ^1^* | | *Overall^1^* | *Contr. ^1^* | *Int. ^1^* | |
| Presence of E.  coli | 72 (76) | 39 (78) | 33 (73) | | 22 (23) | 15 (28) | 7 (17) | |
| Number of CFUs |  |  |  | |  |  |  | |
| <3.5 CFU | 23 (24) | 11 (22) | 12 (27) | | 74 (77) | 39 (72) | 35 (83) | |
| 3.5-10 CFUs | 11 (12) | 10 (20) | 1 (2) | | 5 (5) | 5 (9) | 0 (0) | |
| 11-100 CFUs | 32 (34) | 15 (30) | 17 (38) | | 10 (11) | 6 (11) | 4 (10) | |
| 101-1050 CFUs | 29 (30) | 14 (28) | 15 (33) | | 7 (7) | 4 (8) | 3 (7) | |

*^1^*Numbers are N (%)

**Supplementary Table 3*:*** Number of participants recalling the five WHO moments for hand hygiene when asked during which moments they are supposed to perform hand hygiene.

| **WHO five moments for hand hygiene** | **Burkina Faso** | | | **Mali** | | | |  |
| --- | --- | --- | --- | --- | --- | --- | --- | --- |
|  | *Overall^1^*  (n=95) | *Control^1^*  (n=51) | *Intervention^1^*  (n=44) | | *Overall^1^*  (n=105) | *Control^1^*  (n=56) | *Intervention^1^*  (n=49) | |
| **Before touching a patient** | 91 (96) | 50 (98) | 41 (93) | | 89 (85) | 49 (88) | 40 (82) | |
| **Before a medical aseptic procedure** | 46 (48) | 29 (57) | 17 (39) | | 75 (71) | 41 (73) | 34 (69) | |
| **After a risk of body fluid exposure** | 39 (41) | 28 (55) | 11 (25) | | 64 (61) | 33 (59) | 31 (63) | |
| **After touching a patient** | 90 (95) | 50 (98) | 40 (91) | | 89 (85) | 44 (79) | 45 (92) | |
| **After touching the environment of a patient** | 38 (40) | 23 (45) | 15 (34) | | 39 (37) | 18 (32) | 21 (43) | |
| **All of the moments** | 15 (16) | 10 (20) | 5 (11) | | 32 (30) | 13 (23) | 19 (39) | |

*^1^*Numbers are n (%)

#### **Supplementary Table 4:** General knowledge of participants about hand hygiene. Numbers and percentages are given for the correct answers.

| **Question / Statement** | **Burkina Faso** | | | **Mali** | | |
| --- | --- | --- | --- | --- | --- | --- |
|  | *Overall^1^*  (n=95) | *Contr. ^1^*  (n=51) | *Int. ^1^*  (n=44) | *Overall^1^*  (n=105) | *Contr. ^1^*  (n=56) | *Int. ^1^*  (n=49) |
| Visibly clean hands can contain dangerous bacteria (right) | 93 (98) | 49 (96) | 44 (100) | 92 (88) | 48 (86) | 44 (90) |
| If you touch a patient but not their body fluids, your hands remain clean (wrong) | 91 (96) | 50 (98) | 41 (93) | 94 (90) | 50 (89) | 44 (90) |
| If you touch a patient’s objects, your hands remain clean (wrong) | 94 (99) | 51 (100) | 43 (98) | 100 (95) | 53 (95) | 47 (96) |
| Handwashing with soap eliminates bacteria and viruses (right) | 64 (67) | 36 (71) | 28 (64) | 60 (57) | 31 (55) | 29 (59) |
| Alcohol-based handrub is an alternative to handwashing with soap, even if the hands are visibly dirty (wrong) | 63 (66) | 39 (76) | 24 (55) | 68 (65) | 35 (62) | 33 (67) |
| Handwashing with water is less efficient than handwashing with soap and water (right) | 89 (94) | 48 (94) | 41 (93) | 71 (68) | 42 (75) | 29 (59) |
| Handwashing is a part of the health care process (right) | 95 (100) | 51 (100) | 44 (100) | 104 (99) | 55 (98) | 49 (100) |
| Which of the following transmission paths is the main reason for patient contamination in a health care facility? |  |  |  |  |  |  |
| Air circulating in the hospital | 5 (5) | 4 (8) | 1 (2) | 3 (3) | 1 (2) | 2 (4) |
| Exposure of patients to colonized surfaces | 15 (16) | 5 (10) | 10 (23) | 6 (6) | 2 (4) | 4 (8) |
| Hands of health care workers if not clean (right) | 69 (73) | 39 (76) | 30 (68) | 86 (82) | 46 (82) | 40 (82) |
| Sharing of non-invasive objects between patients | 6 (6) | 3 (6) | 3 (7) | 10 (10) | 7 (12) | 3 (6) |
| *^1^*Numbers are N (%) |  |  |  |  |  |  |

#### **Supplementary Table 5:** Demonstrated handwashing steps by health care workers

| ***Handwashing step^1^*** | ***Burkina Faso*** | | | ***Mali*** | | |
| --- | --- | --- | --- | --- | --- | --- |
|  | *Overall^2^*  (n=95) | *Contr.^2^*  (n=51) | *Int.^2^*  (n=44) | *Overall^2^*  (n=105) | *Contr.^2^*  (n=56) | *Int.^2^*  (n=49) |
| 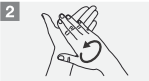Rub hands palm to palm | 85 (89) | 50 (98) | 35 (80) | 91 (87) | 46 (82) | 45 (92) |
| 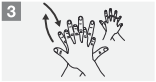Right palm over left dorsum with interlaced fingers and vice versa | 83 (87) | 45 (88) | 38 (86) | 92 (88) | 48 (86) | 44 (90) |
| 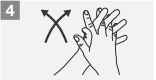Palm to palm with fingers interlaced | 84 (88) | 46 (90) | 38 (86) | 95 (90) | 47 (84) | 42 (86) |
| 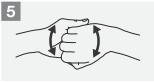Backs of fingers to opposing palms with fingers interlocked | 25 (26) | 17 (33) | 6 (18) | 78 (74) | 41 (73) | 37 (76) |
| 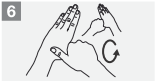Rotational rubbing of left thumb clasped in right palm and vice versa | 59 (62) | 34 (67) | 25 (57) | 75 (71) | 38 (68) | 37 (76) |
| 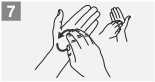Rotational rubbing, backwards and forwards with clasped fingers of right hand in left palm and vice versa | 65 (68) | 36 (71) | 29 (66) | 85 (81) | 45 (80) | 40 (82) |

^1^ Steps 0-1 and 8-11 are not displayed because some participants demonstrated the steps without access to a handwashing facility.

^2^ Numbers are N (%)

#### **Supplementary Table 6:** Behavioral factors underlying handwashing reported on a likert scale from 0-5. Results reported in this table summarize all answers given in the range of 4-5 in the scale.

|  | ***Burkina Faso*** | | | ***Mali*** | | |
| --- | --- | --- | --- | --- | --- | --- |
|  | *Overall^1^*  (n=95) | *Contr.^1^*  (n=51) | *Int.^1^*  (n=44) | *Overall^1^*  (n=105) | *Contr.^1^*  (n=56) | *Int.^1^*  (n=49) |
| Intention to wash hands^2^ | 86 (91) | 46 (91) | 40 (91) | 99 (94) | 52 (93) | 46 (95) |
| Effort to collect water | 37 (39) | 16 (31) | 21 (48) | 14 (13) | 7 (13) | 7 (14) |
| Effort to always wash hands^2^ | 30 (32) | 11 (22) | 19 (43) | 14 (13) | 7 (13) | 7 (14) |
| Frequency of forgetting to wash hands^*^ | 11 (12) | 6 (12) | 5 (11) | 8 (8) | 5 (9) | 3 (6) |
| Barrier: time | 14 (15) | 4 (8) | 10 (23) | 4 (4) | 3 (5) | 1 (2) |
| Barrier: slow water | 5 (5) | 2 (4) | 3 (7) | 3 (3) | 2 (4) | 1 (2) |
| Barrier: no soap | 9 (9) | 3 (6) | 6 (14) | 2 (2) | 1 (2) | 1 (2) |
| Barrier: no water in station | 10 (11) | 4 (8) | 6 (14) | 0 (0) | 0 (0) | 0 (0) |
| Barrier: station not functioning | 15 (16) | 5 (10) | 10 (23) | 2 (2) | 1 (2) | 1 (2) |
| Amount of team members washing hands^2^ | 65 (68) | 35 (69) | 30 (68) | 96 (91) | 52 (93) | 44 (90) |
| Degree of personal importance to wash hands^2^ | 89 (94) | 47 (92) | 42 (95) | 104 (99) | 55 (98) | 49 (100) |

^1^ Numbers are N (%)

^2^ during the WHO five moments for hand hygiene (before touching a patient, before an aseptic/medical procedure, after body fluid exposure, after touching a patient, after touching the patient environment)
